# Exploring how mindfulness enhances attentiveness: a qualitative study with South African mental health nurses

**DOI:** 10.1186/s12912-025-03008-w

**Published:** 2025-05-20

**Authors:** Rudo Ramalisa-Buḓeli, Emmerentia du Plessis, Suegnét Scholtz

**Affiliations:** 1 University of Pretoria, Pretoria, South Africa; 2 North‒West University, Potchefstroom, South Africa; 3 North‒West University, Vanderbijlpark, South Africa

**Keywords:** Attentiveness, Mental health nurse, Mindfulness, Nurse–patient relationship

## Abstract

**Background:**

This qualitative, constructivist grounded theory addressed the gap in understanding how mental health nurses cultivate attentiveness through mindfulness practices. This was done by examining attentiveness as both an expression of care and a facilitator of human connectedness.

**Design:**

Constructivist grounded theory.

**Methods:**

The study population consisted of nurses working in South African psychotherapy wards, where mindfulness was integrated into daily care practices. Three psychiatric hospitals in South Africa were selected, and 11 participants were recruited via nonprobability snowball sampling method. The data were collected between June and November 2021 via virtual and face-to-face individual interviews supplemented with field notes.

**Findings:**

Three categories of cultivating attentiveness through mindfulness practices were identified: foundations for fostering attentiveness through mindfulness; mindfulness practices; and the outcomes derived from such practices. These categories exhibited interconnectedness by featuring shared ideas and overlapping themes and subthemes.

**Conclusions:**

When mental health nurses in this study practice mindfulness, they are attentive and develop a deeper understanding of themselves which conveys to patients.

## Background

Apart from various practical aspects of nursing, professional work raises important questions concerning moral and ethical responsibilities. Among these, attentiveness is a pivotal component of healthcare and assumes a complex role in nursing practice [1]. Rooted in an understanding of the concept, attentiveness encompasses a commitment to perceive and comprehend the intricate processes of care and patients’ needs. In nursing, attentiveness extends beyond observation. It involves deliberate and empathetic engagement with patients, where nurses not only attend to physical ailments but also recognize the emotional, psychological, and social dimensions of the patient [2].

*Attentiveness* is the core element in care and can be understood as a necessary ‘way of acting’ or ‘being’ to know (or to help) other people [1] and is defined as the ‘quality of individuals to open themselves to the needs of others.’ The concept of attentiveness has a clear vernacular and conceptual meaning and has been investigated in many research studies (including caring [2–4], theories [5, 6] and caring presence anecdotes [1, 7–9]. It goes beyond simply being present with a patient but includes genuinely understanding a patient’s verbal and non-verbal communication, emotional states, and personal context. Attentiveness contributes to building a therapeutic alliance, fostering trust, and promoting an environment where patients feel safe and understood. However, despite its importance in professional healthcare practice [1], attentiveness remains to be considered in the nursing literature.

The ethical dimensions of attentiveness have been discussed within the ethics of care literature. Scholars such as Carol Gilligan (2016) and Nel Noddings (1999) have laid foundational work in this field, arguing that caring is a relational and morally significant practice that requires an open and empathetic stance toward others’ needs. Similarly, Joan Tronto’s [6] work on the ethics of care offers another point for understanding this concept because care and attentiveness are internally connected and essential elements of each other [1]. Tronto further proposes that care is a process with four phases (each with a concomitant virtue) comprising attentiveness (“caring about”); responsibility (“taking care of”); competence (“caregiving”); and responsiveness (“care-receiving”). Furthermore, Tronto stated that, from a theoretical perspective, attentiveness is the first step in this caring process [6].

Being attentive resides in paying attention to the processes of care as well as the receiver of care [6]. Nurses who apply all four dimensions of the ethics of care principles in their practice provide the kind of care that is preferable or even superior to mainstream care delivery [10]. Such care is not only technical but also social (“attentiveness”), which is often not recognized [1, 11]. The social side of care allows nurses to have perceptions of the need of patients [10]. In other words, the needs of patients are the initial stimulus in care; however, without nurses’ constant attentiveness, these needs may not be met [6].

There is a need for an attentive approach in the South African health care system, especially considering the nursing landscape and the context within which they practice. Simultaneously, there is an urgent demand for nurses to reshape the image of nursing and to “reconstruct and revitalize the nursing profession for a long and healthy life for all South Africans” [12]. Nurses have been described as inhumane, unprofessional, demotivated and incompetent [13]. This stance is illustrated in the following example where Human Rights Watch [14] interviewed women who had experienced physical and verbal abuse in the hands of nurses and public health workers in hospitals. These women described having been pinched, slapped and handled roughly during labor. They further explained delays in treatment and being ignored by nurses when calling for help.

Mental health care services are also marred by numerous incidents that affect the care nurses provide to patients. In 2018, the Citizen newspaper reported on nurses who locked a mentally ill patient in a seclusion room, and he was found unattended with burn wounds [15]. In another article, it was reported that nurses were not perturbed by a mental health patient who walked nakedly around the wards and hurled insults at other patients [16]. These reports on the treatment of mental health patients exacerbate other challenges facing mental health service delivery in South Africa such as the changing landscape of mental health after the COVID-19 pandemic.

Mental healthcare patients receive care in contexts within which controversial, conflicting and coercive measures are often applied. Such measures can include forced administration of psychotropic medication against the will of patients, involuntary admission in isolation or seclusion, and manual or mechanical restraint to prevent free movement following aggressive behaviour [17]. This paints a picture of the psychiatric unit antitherapeutic to mental healthcare patients via rigid ward rules rather than a therapeutic milieu that facilitates healing [18, 19]. Therefore, nurses working in mental healthcare are in a unique position to display attentive behaviour towards these vulnerable patients.

Drawing on the call for nurses to be attentive in care may present a challenge because of today’s fast-paced healthcare [20], which is favoured by cost-cutting measures [21]. Furthermore, high patient and low nurse ratios diminish nurses’ ability to be present with the patient. According to Drahošová and Jarošová [2], the nurse‒patient ratio and the context within which nurses care influence the time they dedicate to each patient’s care. The relationship and connection between the nurse and patient, which is a fundamental focus of attentiveness, is thus distorted [1, 22, 23]

Nurses can express attentiveness through the sincere desire to listen and help [2, 20]. This desire to listen to and understand patients implies that nurses must have self-awareness and self-acceptance of their personal experiences [20]. Self-awareness and caring go hand in hand to enhance the nurse‒patient relationship and can be fostered by mindfulness practices as nurses reflect inwardly to improve mutual understanding, trust, compassion and clinical outcomes [24].

There is a noticeable gap in the nursing literature concerning comprehensive exploration and the theoretical underpinning of attentiveness. Taking into consideration the challenges faced by South African nurses and the landscape of the healthcare system, the researchers identified that they cannot conclude whether existing research into attentiveness may be transferable or generalizable to the South African context. This is especially the case in the context of mental healthcare, which has multifaceted challenges. This qualitative study was motivated by the need to gain an in-depth understanding of how nurses in mental healthcare cultivate attentiveness through mindfulness practices. Therefore, we aim to explore how mindfulness practices enhance the attentiveness of nurses working in mental healthcare. We further seek to address the question; how does mindfulness enhance attentiveness of mental health nurses?

We underpinned this research on Pierre Bourdieu’s practice theory, which examines how social actions are influenced by the interaction between personal dispositions (“habitus”), the surrounding social environment (“field”), and resources or forms of power (“capital”) [25]. This theory provides a lens through which developing a practical strategy to examine the factors influencing the caring behaviours of mental healthcare nurses. Habitus is structured by past and present circumstances; this structuring is used because it helps shape present and future practices and is formed through personal and professional experiences. As it is systematically ordered, habitus can be regarded as a structure [25]. In the present research, habitus was highlighted through understanding the past and present personal experiences and circumstances in the lives of the nurses.

## Methods

This constructivist grounded theory study was the first phase of a comprehensive three-part research project conducted as part of the corresponding author’s Doctor of Philosophy degree. In the main study each phase had a specific objective which informed the subsequent phase. The first empirical phase aimed to generate and formulate a substantive theory on how nurses cultivate attentiveness through the application of mindfulness practices. Phase two was to use the findings of phase one to develop a model that will conceptualise a practice framework that cultivates attentiveness in nurses working in mental healthcare. Lastly, phase three’s objective was to describe the recommendations for the operationalisation and evaluation of the model.

The study population comprised nurses who were working in psychotherapy wards that provided treatment and different therapeutic modalities. These modalities provided to mental healthcare patients (in South Africa, referred to as mental healthcare users) included dialectical behavioural therapy (DBT) and mindfulness practices. For a nurse to be included in the study, they had to incorporate mindfulness into their daily practice. Three psychiatric hospitals situated in two provinces in South Africa were selected, and 15 participants were sampled via the nonprobability snowball sampling technique. In total, 11 participants consented to participate.

Early in the research, key informants were nominated by mediators, and follow-up participants were nominated by participants who had completed their interviews. These interviews were individual. The latter interviews were carried out as the process of emergence evolved and the theory developed. This allowed the researcher to move on to theoretical sampling [26, 27] and therefore enabled the researchers to develop a theory that is grounded in real-world data by building on the codes that are gathered in follow-up interviews.

## Data collection and analysis

Data collection took place between June and November 2021 through virtual and face-to-face individual interviews depending on the level of COVID-19 lockdown restrictions the country was under. In other words, during “hard lockdown”, the interviews were virtual (n = 5), but once the restrictions had been lifted, face-to-face (n = 6) interviews were conducted. Each interview was between 45 and 60 minutes in duration and were audio-recorded. Field notes were also taken during the interviews for further comparison and supplement recorded data. The corresponding author transcribed the audio-recorded interviews verbatim.

Using semi-structured interviews allowed for an evolving inquiry where subsequent interviews were adapted to probe the emerging categories in depth. Which aligns with Corbin and Strauss’ [28] recommendations to continually refine and densify emerging categories.

The initial interviews were broad and exploratory and focused on participants’ understanding of attentiveness, personal experiences and how mindfulness influenced their work were conducted in a semi-structured format with a guide for the structured questions (see below). However, as the research progressed, later interviews were tailored to explore the emerging codes in greater depth.

- Nurses with a high level of attentiveness in care have the potential to improve the care experience of patients. What is your understanding of attentiveness in care?
- Tell me of a time or incident in your life when you felt that someone is being attentive to you.
- If at all, how do you think mindfulness practices have contributed to how you provide care, especially to mental healthcare users?

**Fig. 1:**
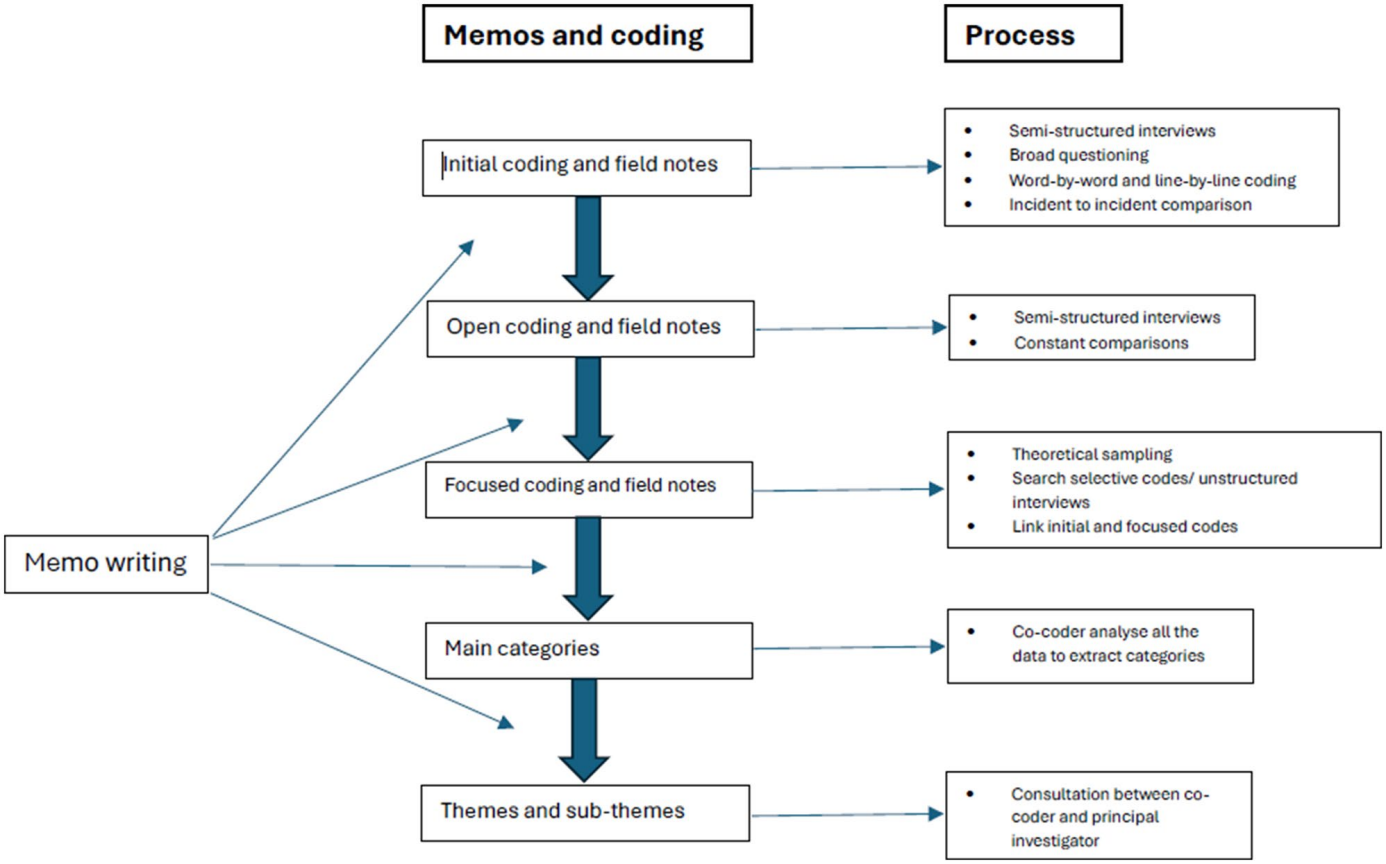
Diagrammatic representation of data collection and analysis

As the codes and categories began to emerge, subsequent questions were more targeted and specific. Figure 1 illustrates a diagram of how the questioning evolved during the study, showing the initial broad focus and tailored questions later.

Analysis of the data began immediately after each interview, and memos were written to document the researcher’s thought, questions and identify emerging patterns. Similarities and differences were identified across the interviews through constant comparison to allow for categories and themes to develop.

In the initial analysis, the data revealed personal awareness and attunement to patients as prominent codes with further interviews delving in depth into these codes and other arising codes. The goal was to ensure that each arising code and category was well developed in terms of density and variation [28] and that the direction of the grounded theory research was determined by participants.

As outlined by Corbin and Strauss [28], it is recommended that researchers formulate a strategy for analyzing the data. In line with this, the principal researcher adopted a strategy of deconstructing the narratives provided by participants, reassembling them, and discerning distinct categories and their associated properties by posing incisive analytical inquiries concerning the data [28].

Rigour was enhanced by employing an independent coder whom the principal investigator explained the data collection method and analysis. They were handed the transcribed interviews in chronological order of interview, the analysis protocol, and the principal investigator’s memos. Collaboratively the principal investigator and coder discussed any discrepancies to refine the codes.

Using the constructivist grounded theory was important to this study as it provided a systematic and flexible method to explore and co-construct the topic. The data collection and analysis process which involved constant comparison and theoretical sampling ensured that the codes and categories were grounded in the participants’ experiences [28].

## Ethical considerations

This study is part of the “caring presence” research niche within the NuMIQ (Quality in Nursing and Midwifery) focus area at the North-West University’s School of Nursing Science. The North‒West University Health Research Ethics Committee granted full ethical approval for this research (ethics number NWU-00494-20-A1). Prior to obtaining this approval, permission was first secured from the participating hospitals. Hospital management, with support from the two Provincial Departments of Health where the study was undertaken.

The research involved human participants; therefore, the research procedures were conducted in accordance with the Declaration of Helsinki [29]. And ethical guidelines and regulations set out by the National Health Research Ethics Council of South Africa [30] were followed. Before taking part in the study, all participants provided informed consent after receiving information outlining the research objectives, potential benefits and risks, privacy considerations, voluntary participation, and the right to withdraw. Data collection began only after securing both ethical approval and necessary permissions.

## Results

The study participants included 11 registered nurses. Nine were actively working in psychotherapy wards across three psychiatric hospitals in South Africa at the time of interview and 2 had recently resigned. Participants varied in years of experience, ranging from 5 to 20 years in the nursing field. The group included one male and ten female nurses, with an age range of 26 to 55 years.

Three primary categories were identified, namely, foundations for fostering attentiveness through mindfulness; mindfulness practices; and the outcomes derived from such practices (see Table 1). These categories exhibit interconnectedness by featuring shared ideas and overlapping themes.

**Table 1:** Cultivating attentiveness through mindfulness practices

Categories	Themes	Sub-Themes
Category 1: Mindfulness practices	Theme 1: Mindfulness practices and developing embodied qualities	
	Theme 2: Qualities of being mindful	
Category 2: Outcomes of mindfulness practice	Theme 1: Being attentive to self and others	Sub-Theme 1: Attentiveness heightens the mental healthcare nurse’s level of awareness
		Sub-Theme 2: Attentiveness contributes to a holistic care approach
	Theme 2: Mindfulness enhances mental healthcare nurses’ mental well-being	
	Theme 3: Mindfulness benefits the multidisciplinary team	
Category 3: Foundations for cultivating attentiveness	Theme 1: Mindfulness creates an awareness	Sub-Theme 1: Being aware of the self
		Sub-Theme 2: Being aware of the mental healthcare patient
	Theme 2: Setting boundaries	
	Theme 3: Specific requirements needed for being mindful	Sub-Theme 1: Teachable and learning moments
		Sub-Theme 2: Support systems

## Category 1: Mindfulness practices

The participants alluded to mindfulness practices through which they developed qualities such as calmness, patience, trust, and active listening. Mindfulness was reported to be integrated into the participants’ workplace. This enabled participants to make a conscious choice to embody the qualities and develop attitudes and behaviors that cultivate their attentiveness in care. Two themes were classified under this category and are discussed below.

### Theme 1: Mindfulness practices and developing embodied qualities

Mindfulness practices undertaken by the participants included self-soothing meditative exercises, body scans, mindful breathing, and taking a mindful walk. Their experience with mindfulness practices was seen as enabling them to feel contained and grounded and allowing them to bring their complete attention to the present moment. The participants were able to listen actively and respond, demonstrate compassion, and observe situations or patients with a nonjudgmental attitude. As observed by P3:

I mostly practice meditation as a form of mindfulness to help me stay grounded, even if I am going through many things and I feel like I have to manage a lot that I am dealing with; to stay grounded, I use meditation a lot. (P3)

P4 noted, “We do the breathing exercise with the patient, trying to ground them. Asking them and exploring what happened before the patient reached this state.” P5 proposed the following: “Maybe do something with them or go for a walk, do breathing stuff like that.” P11 offered the following suggestion: “You know about grounding and breathing, so let us do that. You know how you can [sic] self-soothe in this moment, as you are feeling very emotionally aroused.”

### Theme 2: Qualities of being mindful

The cultivation of specific qualities among participants was credited to the mindfulness practices practiced by nurses. These qualities included adopting a nonjudgmental stance, directing attention toward the present moment, acknowledging personal strengths and weaknesses, fostering empathy, exercising patience, engaging in self-soothing and calming techniques, and approaching active listening with unwavering intent.

Being mindful was seen as nurturing caring behaviors, including sympathy and empathy. This was observed through the participants’ reports that cultivating self-compassion played a role in revealing innate compassionate attitudes toward mental health patients. Finally, mindfulness was also reported to contribute to the demonstration of reassurance. Reassurance was viewed as necessary to calm the patient down, as this serves to alleviate the anxiety and stress frequently experienced by mental health patients. P3 noted, “What I think I have experienced with mindfulness is learning to stay calm and realize, pick up, and notice my emotions and notice my feelings that could come up, through all of that I could remain calm myself.” P4 offered the following: “It affected me in calming myself and helped me to think straight. Before I say something, I know I need to think about it.” P6 declared, “It is so easy to put that judgement. We forget that we are here to nurse. We label the person. You have a label and now we want to nurse you according to your label.” P7 shared, “Because in this kind of ward, you need to have, like I’ve mentioned before, sympathy and empathy with this kind of patient.”

P9 reflected:

How I recognize my strengths and my weaknesses thinking and doing some introspect to see that, well, this is what I took away from the situation.… self-introspection and self-reflection is very important because then what you don’t want to do is then intertwine your life with the patient’s life and then find similarities and say oh but yeah, actually you know what I was also in the same situation, and this is what happened in my situation in my experience. (P9)

## Category 2: Outcomes of practicing mindfulness

The participants noted enhancements in various facets of their professional experiences.

### Theme 1: Being attentive to the self and others

Moment-to-moment attentiveness was reported to be strengthened when individuals consistently engaged in mindfulness practices. This was seen to foster heightened awareness and to provide an especially focused presence, including in participants’ daily experiences. Thus, they experienced heightened awareness that enabled them to notice and acknowledge the struggles of mental health patients. This theme came about when we noted that participants described how they perceived themselves in relation to the patients. In other words, participants saw themselves through patients, and thus, a personal nurse–patient relationship was formed. Thus, mental healthcare nurses were observed to treat patients courteously and as humans rather than merely “treating the illness”.

#### Subtheme 1.1: Heightened level of awareness

Attentiveness to patients was described by referring to the behavior of mental health care nurses. The participants described how they could take notice (“be aware”) of changes in the patient’s condition because they practiced open attentiveness when interacting with patients. Noticing changes involved using their senses to detect what was happening in the present moment. When the changes had been noticed, the participants further made the patient aware of the observed changes. In turn, this made the patients feel cared for and gave them the courage to practice mindfulness skills as well.

At the same time, the nurse taking notice of subtle behavioral or emotional changes indicated attentiveness, and the patient felt validated. This was identified in the responses of the participants. P1 stated, “If you are quite attentive, you can note things quicker if there is a change in a patient.” P3 offered the opinion that “being able to pay attention to what is happening in the present moment [is important], so paying attention involves me using my senses, looking to what the patient is doing, listening to what the patient is saying.” P6 observed:

If you don’t be that active listener and by active listening, is observing the patient while you are talking. I notice you are anxious, or I noticed in the beginning of the conversation you were like this and this and this. Did you notice there was a change from how you walked in and how you walked out? (P6)

As expressed by P8, “The patients sometimes find it as a sense of caring and a real interest in them, if you notice something changed about them. Then, they feel more cared for because this person picked up that something is wrong.” This first subtheme of category 2 supports changes in behavior through open attentiveness.

#### Subtheme 1.2: Attentiveness contributes to a holistic care approach

The participants shared that the direction of their interaction was often led by patients; for example, participants indicated that when a patient initiates a conversation about religion and spirituality, the nurse would subsequently confirm their comfort with such topics. Furthermore, the participants used concepts such as “patients are like family”, “acceptance” and “patients must trust you” to describe attentiveness. Comparing the patient to the family is suggestive of an alliance that plays out between the nurse and patient because the family is paramount in expressing care experiences and relationships.

Finally, the participants described how they provide “tailor-made care” for individual patients’ needs. In this case, the patient’s willingness to practice the mindfulness skills taught to them in the ward and embody those skills because they feel valued implies holistic care, which reflects the well-being of the patient. As stated by P2, “Proper care is holistic. It just needs to be holistic. I think the trick here being a nurse therapist is that as much as you are the one that challenges the patient when they come to say I have got a headache.” P5 thought that there was a need to “care for human beings holistically, not only basic needs but even mentally, spiritually.” P7 expressed the view: “We have to treat them with respect. See to their basic need, see to their emotional needs as well.” As noted by P8, “The care is actually holistic care. Even though it is psych [sic], we also focus on the physical side of the patient. Very, very warm care.”

In essence, the participants highlighted that being attentive pertains not only to the duration of the time that the mental healthcare nurse spent with the patient but also to the quality of the time spent with the patient.

### Theme 2: Mindfulness enhances the mental healthcare nurse’s mental well-being

By establishing frequent mindfulness practices, the participants reported that their well-being had improved. The participants considered mindfulness practices to be tools that enabled them to stay grounded. In turn, this allowed them to have constant awareness of their emotional well-being and how their emotions fluctuated throughout the day. P3 expressed the following opinion: “Being able to stay grounded in the present moment, I think it is something that would be helpful to us as healthcare professionals.” P8 made the following observations:

However, I think self-care should be one of the nurses’ priorities. In addition, it’s not by dressing up it’s also with your mind. We should make time for ourselves; even if you go out to the shops, you spend the day on the beach alone, exercising will help because it’s good for the mind; it is good for the body. We tend to be so trapped in our daily routine that we don’t make time for things like that. Therefore, just taking a 10- to 15-minute walk would be appropriate. (P8)

P11 shared the following:

I am feeling a bit stressed; I take a deep breath, and that happens throughout the day. Because you know I am doing so many different things at one time. You know, and there is so few of us also, so I do find that I have to ground myself in that way. (P11)

### Theme 3: Mindfulness benefits the multidisciplinary team

As mindfulness was integrated into the ward setting, all members of the multidisciplinary team might benefit; the resonating sentiment for this theme was that attentiveness could seamlessly permeate the entire treating team. When working with (in) a team consisting of various team members, each member had a specific role and brought a different dynamic to the patient treatment process. The participants stated that they were not only attentive to providing care but also attentive to their colleagues, other members of the multidisciplinary team and students. As observed by P1:

It helps to have a team to work with, it helps to talk about everything that happens with the patient and then with the team you sit in a mindful way and discuss that okay, no I think you are overstepping. (P1)

P2 shared the following:

Because I am able to not only be attentive to patients but also to the students I have in the ward, I am attentive to other staff members, the multidisciplinary team in a sense and the other nursing staff. Therefore, it benefits the team as a whole. Because I am also able to help other nurses process what is happening in the ward. (P2)

Furthermore, P4 shared the following:

I know what to do in situations and are also able to accommodate my colleague as well in sharing the knowledge that I have. In addition, to contribute to the treating team as well and confidently so say, I am part of the team. (P4)

## Category 3: Foundations for cultivating attentiveness

The participants considered discourses that established a foundational principle and necessitated the cultivation of attentiveness among mental healthcare nurses. These premises were enhanced and developed through sustained engagement with mindfulness practices. In other words, the conscientious practice of mindfulness serves to strengthen and enforce the nurse’s attentiveness and care. This approach was developed when nurses attained heightened awareness of both them and others, set boundaries, maintain an effective support system and engage in teaching and learning. These premises either needed to be in place at the outset, or the nurses developed or enhanced them when practicing mindfulness.

### Theme 1: Mindfulness creates awareness

Two distinct forms of awareness became apparent under this theme: (a) self-awareness and (b) awareness of mental health care patients. Mindfulness intensified self-awareness to better understand their own and others’ emotions, while it strengthened mental health care nurses’ ability to direct their awareness toward patients’ struggles and needs.

#### Subtheme 1.1: Being aware of the self

The participants noted that self-awareness positively impacted emotional maturity, self-confidence, and self-esteem. The participants reported that working in the psychotherapy unit was challenging; some nurses were reluctant to work in the unit because they perceived a risk that they would absorb negative energy. The recognition of strengths and weaknesses influenced the responses. Self-awareness was found to be crucial for sustained attention in nursing. This awareness extends beyond egoistic self-reflection; rather, it serves as a relational safeguard. Nurses were identified as needing to be vigilant about conduct that affected mental health care patients. The participants also highlighted that applying mindfulness skills while interacting with patients advanced their awareness of their state of mind in the (“moment-to-moment”) here and now.

P5 expressed these sentiments: “Emotionally, it made me being (sic) able to handle all the stress or emotional stuff that was happening to me like. Self-awareness, yeah, and even my self-esteem my confidence like also I could stand up for myself.”

P3 further noted the following: “To know yourself as an individual so that you can be helpful to someone else, either it be a patient, friend, or a family member. Being aware of yourself helps in the case of mental health being a nurse and rendering care.”

The following was conveyed by P11:

Whether we realize or not, patients pick up through subtle body language and things that I don’t even know I might be projecting. They will pick it up, so that is why I am always aware. Every little thing, every slight, every facial expression that you have, they take personally. Therefore, I truly have to be mindful of that. A lot of the time, like how is my tone, what does my face say you know? Because they just watch everything. (P11)

#### Subtheme 1.2: Being aware of mental health care patients

The second awareness that stemmed from practicing mindfulness was that nurses could be aware and truly connect with the patients they were caring for. The mental health care nurse acknowledged and accepted the mental health care patient for who they were. This acknowledgement was achieved by demonstrating a genuine interest in the patient, where they felt valued. This genuine interest also extended to comprehending the patient’s experiences through active acknowledgement and reassurance. P2 emphasized that “There is a better sense of acknowledging the patients for who they are and giving the same level of respect you would give to any other patient.” While

P9 stated the following: “So being able to get to the patient’s level and understand the current circumstances, but the circumstances that are surrounding them and their illness. Therefore, I’ve always kept that in mind.”

P10 also noted that Listening to the patient at least helps you to see that OK, this type of patient on this type of day may run a type of principle of a mindfulness group about kindness to self and others. This patient struggled after the group because most of the patients worked, but there was a certain principle (group) that they struggled with. (P10)

### Theme 2: Setting boundaries

The second theme in this category focused on the boundaries that had to be established for optimal care. These findings suggested that these boundaries allowed nurses to remain connected with their own emotions and well-being when confronted with challenging encounters, ensuring that they did not create unnecessary distance from the patient. This distance hindered the delivery of the optimal provision of care.

The participants reported that they developed professional boundaries, ensuring that they effectively compartmentalized their feelings when providing care. This compartmentalization of emotions is a vigorous process of creating a mental space to be mindful. Some participants reported that they can “feed off” and take on patients’ emotions.

Mindfulness also resulted in participants recognizing and embracing their personal experiences, which enabled them to sympathize. Certain feelings or behaviors are triggered when patients tend to share their traumatic experiences, which may negatively affect the care process. However, in this subtheme, the participants also indicated that being mindful helped them prevent their thoughts from dwelling excessively on their past experiences. P1 described the following:

Sometimes patients have gone through the same backgrounds you have gone through. In addition, then if you are not mindful that this is the situation, so how do I separate my past, it’s not about me right now; it’s about my patient. (P1)

P8 also echoed the following sentiments:

Because you experience the same trauma that they experienced, especially if you start to become very close to your patient. Therefore, you sit and then you truly feel what you think what that person was going through at that time. (P8)

Similar sentiments were also expressed by P9: “We are bound to have certain experiences that we share the same things with, but it’s also finding the clear line that look you cannot speak of your experiences because what you don’t want is to invalidate someone else. Your experiences help you to better understand and better empathize with the patient but do not take away the moment from the patient. (P9)

Last, P11 was also related to patients’ experiences: “There have been times that I was able to relate to the patient, maybe we have been through similar experiences, but I’m still able to maintain that mental boundary quite firmly.”

### Theme 3: Specific requirements needed to be mindful

The two subthemes in this theme were the attributes of being mindful, which included teachable and learning moments, and the need for support systems to be in place.

#### Subtheme 3.1: Teachable and learning moments

The participants reflected on how teaching and learning mindfulness skills is a requisite for cultivating attentiveness. The development and full embodiment of mindfulness skills was identified as a gradual process, which required the willingness of mental healthcare nurses. However, once the skills were mastered, nurses also became open-minded to learning. Mental healthcare nurses developed greater awareness of themselves, and embodying mindfulness practices also required ongoing implementation. The participants indicated that in their personal spaces, they had time to incorporate some of the practices. Learning also takes place through mentorship and guiding one another in the ward.

The participants also reflected on the impact of teaching and learning on their self-confidence; this was observed among the more experienced nurses in that they had conviction and were skilled. The following was shared by P1:

I think it takes a lot of practice and a lot of learning. Mindfulness should be a subject in nursing training from the first to fourth years. Because not just psych (sic) needs mindfulness, I think each and every discipline in nursing needs mindfulness. (P1)

Self-development was also expressed in the sentiments shared by P2, who stated that I had to equip myself with the appropriate knowledge of the content of what is happening in this ward. Therefore, I personally invested in reading many articles. Reading up a lot of content regarding where I am going. (P2)

P4 mentioned the following: “and self-improvement and knowing my job. To respect my job as well and have that knowledge and experience in what I am doing.”

#### Subtheme 3.2: Support systems

Some participants frequently lamented the inadequacy of a support system in their respective workplaces, as this influenced how they provided care, whereas some felt sufficiently supported in the workplace by the multidisciplinary team and fellow nurses.

The participants sought help from other colleagues and talked openly about their challenges. To foster this type of support, the relationship needed to be built on trust, as some participants felt that sharing their intimate challenges with their colleagues entailed being discussed in a manner lacking professionalism. This also required having unit managers whom the nurses could talk to. According to P4, “We have supervision once a week, where I take my difficulties there”. P8 also expressed the following regarding support in the workplace: “I think for us it is very important for nurses to have a support structure. Whether it is another nurse or a nurses’ group or if you are having a debriefing session among each other”. P11 also indicated, “I talk openly about it with my colleagues, and we just sit there and support each other, which is really nice.”

## Discussion

This paper highlighted how mindfulness practices contribute to cultivating attentiveness in mental healthcare nursing. The findings emphasize the importance of mindfulness in enhancing nursing presence, self-awareness, emotional regulation, and the nurse-patient relationship. We identified three key categories related to the cultivation of attentiveness namely: mindfulness practices, outcomes of mindfulness, and foundations for cultivating attentiveness. The synthesis of these categories demonstrates that mindfulness practice enables nurses to be more attentive, responsive, and empathetic, while maintaining a deep connection with themselves, patients and colleagues.

When nurses in this study engage in mindfulness practices, which include body scanning, breathing exercises, and mindful walking, their ability to remain present and “be with” patients in their interactions is enhanced. One attribute of this enhancements is a result of neural pathways changes in the brain [31] as a result of frequent practices.

Participants described in category one that by engaging in these practices, they were able to cultivate qualities such as calmness, patience, trust, and active listening, all of which foster attentiveness in care. This quality allows nurses to respond more mindfully to patients, particularly in the high-stress environment of mental healthcare. Beach et al [32] stated that mindfulness is associated with more patient-centred communication, greater rapport building, and a more positive emotional tone during patient interactions. This suggests that mindfulness could enhance qualities such as attentiveness and empathy, which are beneficial during patient care.

Bernstein [33], argued that mindfulness helps nurses accept situations nonjudgmentally, with compassion and curiosity. Similarly in this study, participants in Category 1 and 2, reflected on this stance that they take genuine interest and are compassionate about their patients. Furthermore, participants reported that mindfulness practices not only improved their ability to be present with patients but also helped them regulate their own emotions and stress. These finding give weight to Beach et al [32] who reported that mindfulness practice is linked to a positive emotional tone amongst healthcare practitioner which not only make them attentive listeners but allows their patient to express their psychosocial challenges as well. Becoming grounded through mindfulness is crucial in mental healthcare, where nurses must be attuned to the patient’s needs while managing their emotional boundaries. This is because it is widely established that nurses at the forefront of mental health care, who encounter patients with trauma, violence, anxiety and depression may themselves report emotional burnout [34], and other negative consequences.

Another significant outcome of mindfulness practice, which most participants reflected on, is being present in moment-to-moment interactions and was described as heightened awareness. In this study, participants reflected on self-awareness and their awareness of others. According to Prince-Paul and Kelley [35] awareness is deemed crucial in the caregiving process, as it allows nurses to respond by bringing complete attention to the experiences occurring in the present moment in a nonjudgmental and accepting way with openness and curiosity. Furthermore, they can discern patient needs without interference from their own emotions or judgments. This self-awareness also improves nurses’ ability to recognize nonverbal cues from patients, contributing to a deeper understanding of their emotional states.

From a mindfulness perspective, Klaver [1], stated that the concept of attentiveness lies in the aspect of openly observing. This means that the nurse break through the patterns of normal observations and recognise the patient. Furthermore, attentiveness is a two-part practice: first, getting to know others in their uniqueness, and second, learning how to respond to them effectively while establishing a genuine interest or relation. In this study, nurses reported that mindfulness helped them to better understand patients’ needs, to foster a therapeutic relationship based on mutual respect and trust. This attentiveness also leads to intra- and interpersonal transformation for both nurses and patients, enhancing the overall caregiving experience [20].

Nurses in this study attributed a change in perspective, where they are actively receptive and understand the world from the patient’s viewpoint to mindfulness practices. As noted by Kabat-Zinn [36] and Prince-Paul and Kelley [35], this receptivity cultivates compassion, patience, and empathy. These attributes are important to developing the nurse-patient relationship. Nurses who are present and attentive in this way can respond to patients’ needs more effectively, providing individualized care that reflects an understanding of the patient’s unique experiences.

This study also found that mindfulness practices impact the work environment positively by fostering collaboration and empathy among colleagues. Participants reflected on attunement to their coworkers which has been proved to contribute to a shared sense of purpose and common goals (37). Within mental healthcare this can be especially important, where the emotional demands of the job can create stress and affect communication. This stance was further elaborated in category three on discourses foundational to cultivating attentiveness among mental healthcare nurses, emphasizing workplace teaching and learning as well as the prioritization of support systems.

Our study aimed to explore how mindfulness practices enhance the attentiveness of nurses working in mental healthcare. As attentiveness is an essential component of care [1, 6], cultivating this quality through mindfulness can be deemed as an intervention which may improve patient outcomes through various mechanisms. Such attentiveness facilitates healing, not only by addressing physical and psychological needs but also by cultivating a space for existential and spiritual well-being [9]. By integrating mindfulness practices, nurses may enhance their ability to engage holistically with patients, thereby reaffirming the centrality of human connection in achieving optimal health outcomes. This integration highlights the potential of mindfulness as a transformative tool in mental health nursing.

## Clinical implications

The researchers recommend that training and awareness in the form of workshops and seminars on attentiveness (caring presence theory) should be done with nurses in the workplace, to make them aware of its impact on nursing care. Emphasizing mindfulness practices in the workplace could potentially improve the well-being of mental health nurses and the multi-disciplinary teams by enhancing self-awareness strategies, communication, and creating a more supportive work environment.

## Limitations

This study was conducted in selected metropolitan areas within two South African provinces. The findings are therefore context-specific and reflect the experiences and practices of nurses in these particular settings. Including a broader geographical range with the South African landscape, such as additional provinces, cities or rural areas, may have offered a different perspective in terms of the culture and habitus. However, the insights gained may still be relevant to other similar contexts where mental health nurses work under similar conditions.

The study participant was limited to registered nurses, as other categories were inaccessible. Although we intended to include all the nursing categories in the research, with the workforce being mainly registered nurses in the speciality psychotherapy unit, we were not able to conduct interviews with other categories of nurses. Furthermore, experiences from nurses in other specialties or care settings could provide more understanding of attentiveness.

The research sample size were 11 participants, which, places limits on the conclusion and recommendations as they must be interpreted cautiously and may not be broadly generalizable. However, the findings provide valuable but tentative insight on how nurses can enhance their attentiveness to patient through mindfulness. For generalizability of the findings, the researchers recommend research with larger samples and diverse contexts in nursing.

Another limitation in this study lies in question 1 of the interview questions which states, “Nurses with a high level of attentiveness in care have the potential to improve the care experience of patients. What is your understanding of attentiveness in care?” This question may be seen as leading participants and could influence them to view attentiveness from a positive point. The researchers mitigated this biasness by encouraging and prompting participants to express their genuine views, which include those that do not align with the premise of the question. Also, the follow-up questions in the interview schedule were used to probe and clarify their responses. The remaining questions in the interview schedule were open-ended, allowing participants to freely discuss their experiences.

Finally, the study employed the constructivist grounded theory, findings are limited to the research approach used in this study. The paper presents a small-scale account of a larger study, which may restrict the findings generalizability to a broader contexts or diverse settings. Further research using alternative research approaches such as quantitative methods which may expand on the findings of the present study.

## Conclusions

Before this study, the multiple research projects conducted on attentiveness had not addressed how mental health nurses cultivate attentiveness through the practice of mindfulness. Through the synthesis of three distinct categories, this study highlighted on the integral role of mindfulness in fostering attentiveness and enhancing patient care. By integrating mindfulness into their daily routines and interactions with patients, mental health nurses in this study develop a deeper understanding of themselves and their patients’ needs, leading to more individualized and spontaneous care. This aligns with Kabat-Zinn’s principles of mindfulness-based stress reduction, which emphasize nonjudgment, patience, trust, acceptance, and letting go [36].

Mindfulness also enabled nurses in this study to better understand and respond to patients’ needs but also reduced their anxiety levels and improved their relationships with colleagues. This underscores the broader impact of mindfulness on both individual well-being and interpersonal dynamics within the healthcare environment.

By being mindful of their own emotions, biases, and personal boundaries, nurses were better equipped to provide compassionate and responsive care without being overly influenced by their own experiences. Setting clear boundaries—both physical and mental—allows nurses in this study to create a therapeutic environment which is conducive to patient healing.

This study further extends the existing body of knowledge and addresses the gap in understanding how mental health nurses cultivate attentiveness through mindfulness practices. This study addressed the mental health nursing context given the unique challenges mental health nurses face in high-stress, resource-limited settings, which can impact their ability to remain attentive. In conclusion, this study highlights the transformative effect of mindfulness in cultivating attentiveness among nurses working in mental healthcare; it also enhances the quality of care provided to mental healthcare patients. By fostering self-awareness, presence, and compassion, mindfulness practices enable mental health nurses to sensitively and skilfully navigate complex patient interactions, ultimately promoting positive outcomes for patients and caregivers alike.

## Data Availability

All the data (recorded interviews, consent forms, transcriptions, field notes and data analysis and coding) used in this research are from the corresponding author.
